# Primary succession of ectomycorrhizal fungi associated with *Alnus sieboldiana* on Izu-Oshima Island, Japan

**DOI:** 10.1007/s00572-023-01112-w

**Published:** 2023-05-26

**Authors:** Akira Ishikawa, Kazuhide Nara

**Affiliations:** Department of Natural Environmental Studies, Graduate School of Frontier Sciences, The University of Tokyo, 5-1-5 Kashiwanoha, Kashiwa, Chiba 277-8563 Japan

**Keywords:** Volcanic succession, Alder, *Alpova*, *Tomentella*, Host specificity

## Abstract

The primary succession of ectomycorrhizal (ECM) fungi has been well described for *Pinus* and *Salix*, but the succession for other pioneer hosts is almost unknown. Here, we investigated ECM fungal communities of *Alnus sieboldiana* at different host growth stages in a primary successional volcanic site on Izu-Oshima Island, Japan. ECM root tips were collected from 120 host individuals, encompassing seedling, sapling, and mature tree stages. The taxonomic identity of the ECM fungi was determined based on rDNA internal transcribed spacer region sequences. Nine molecular taxonomic units were detected from a total of 807 root tips. The initial ECM fungal community on the pioneer seedlings was composed of only three species, where an undescribed *Alpova* species (*Alpova* sp.) was exclusively frequent. With host growth, other ECM fungal species were added to the communities, while the initial colonizers remained even at mature tree stages. Thus, the ECM fungal composition significantly changed along host growth stages and showed the nested community structure. Although most of the ECM fungi confirmed in this study had a broad Holarctic geographical distribution, the *Alpova* sp. had no previous records in other regions. These results suggest that a locally evolved *Alpova* sp. plays an essential role in the initial seedling establishment of *A*. *sieboldiana* at early successional volcanic sites.

**Supplementary Information:**

The online version contains supplementary material available at 10.1007/s00572-023-01112-w.

## Introduction

Ectomycorrhizal (ECM) fungi are symbiotic microbes that colonize the fine roots of dominant tree species in temperate to boreal forest ecosystems (Read et al. 2004; Tedersoo 2017). With the supply of photosynthetic products from the host, ECM fungi develop extensive belowground mycelia, which dramatically enhance the water/nutrient uptake of the host plants (Smith and Read 2008). Thus, host plant growth is boosted by ECM colonization, without which the host plants cannot survive in nature. In forest ecosystems, ECM mycelia are so ubiquitous in soil that the roots of existing trees and newly establishing seedlings can be readily colonized by compatible ECM fungi (Simard and Durall 2004; Selosse et al. 2006; van der Heijden and Horton 2009). Even after severe disturbances such as forest fires and landslides that eliminate the existing ECM fungal mycelia, soil spore banks of ECM fungi are so widespread that newly establishing host seedlings can readily find compatible ECM symbionts (Peay et al. 2009; Glassman et al. 2016; Yamanaka et al. 2022). Therefore, ECM formation itself is rarely a problem for host establishment in most forest ecosystems due to the ubiquity of ECM fungal inocula.

By contrast, biological legacies such as ECM fungal inocula are entirely absent in newly exposed substrates produced by volcanic activity, glacier retreat, and sand dune formation at the initial stage (Walker and del Moral 2003). Thus, the establishment of host seedlings is often restricted by the absence of ECM fungi in the early stages of primary vegetation succession. For example, in the volcanic desert on Mount Fuji, the seedling establishment of ECM tree species (*Larix* and *Betula*) was not observed in nearly 99% of the ground area, whereas their establishment was found only in restricted small areas where belowground ECM fungal mycelia were available near pre-established pioneer hosts (*Salix reinii*) (Nara 2006). At this volcanic site, most of the ECM fungi colonizing the pioneer willow were generalists with wide host ranges and thus compatible with the subsequent tree species, enabling ECM colonization and facilitating their establishment. If the pioneer hosts were associated with specialist ECM fungi, as in the case of pine-specific ECM fungi in a sand dune (Ashkannejhad and Horton 2006), they might not directly facilitate ECM colonization of other host trees until generalist ECM fungi are recruited at later stages. Therefore, the composition of ECM fungi colonizing pioneer hosts during primary succession has profound implications for subsequent vegetation succession and forest formation.

Knowledge of ECM fungal communities on pioneer hosts in primary successional sites comes mostly from two host groups; namely, *Salix* and *Pinus*. Pioneer *Salix* is usually colonized by early-stage fungi, including *Inocybe*, *Laccaria*, and *Hebeloma* species (Jumpponen et al. 2002; Nara et al. 2003a, b; Obase et al. 2007). Most early-stage fungi are generalists and compatible with a broad range of host species (Nara 2006). By contrast, ECM fungi on pioneer *Pinus* are often dominated by suilloid species specific to the host or closely related species in the same genus (Ashkannejhad and Horton 2006; Reverchon et al. 2010). In the case of *Salix* or *Pinus*, the composition of ECM fungal communities changes with host growth. Specifically, ECM fungal species belonging to late-stage fungal groups like *Russula* and *Amanita* increase in later host growth stages, probably in association with organic soil development (Dickie et al. 2013). During such primary succession of ECM fungi, the initial colonizers on the pioneer seedlings decrease in frequency and abundance but are still found on mature trees (Nara et al. 2003b; Ashkannejhad and Horton 2006). Unfortunately, we do not know whether such primary successional patterns of ECM fungi can be generalized for all pioneer hosts due to the scarcity of comparable data from other tree genera.

Some *Alnus* (alder) species appear as pioneer tree species in early primary succession areas (Kamijo et al. 2002; Dolezal et al. 2008; Titus 2009), while many other alder species are much more common in riparian and wetland areas in the northern hemisphere (Chen and Li 2004). Unlike other ECM hosts, alder trees can form root nodules with nitrogen-fixing *Frankia* (Chatarpaul et al. 1989; Molina et al. 1994). Thus, alder species can use atmospheric dinitrogen as a nutrient source and grow well in nitrogen-deficient soil, which prevails in primary successional sites. *Alnus* is also known to form symbiotic associations with ECM fungi, which aids phosphorus absorption and host growth (Chatarpaul et al. 1989; Yamanaka et al. 2003; Walker et al. 2014).

Previous studies indicate that ECM fungal communities on *Alnus* are less diverse and dominated by highly host-specific ECM fungi (Molina 1979; Tedersoo et al. 2009; Kennedy and Hill 2010; Bent et al. 2011; Kennedy et al. 2015). In particular, *Alpova* and *Alnicola* are predominantly alder-specific at the genus level (Molina 1981; Moreau et al. 2006). Furthermore, ECM fungal species belonging to generalist genera, such as *Lactarius* and *Tomentella*, can also be specialists to *Alnus* (Rochet et al. 2011; Kennedy et al. 2011; Nouhra et al. 2015), while most other species belonging to these genera can colonize a wide range of hosts. Põlme et al. (2013) reported 146 ECM fungal taxa from 22 *Alnus* species at 96 sites worldwide and found that ECM fungal communities were significantly affected by host phylogenetic relationships even within the genus. However, none of these studies were conducted in primary successional areas, and thus the effect of host developmental stages on alder ECM fungal communities under primary succession remained unknown.

On volcanic islands, *Alpova* species were confirmed in bioassay experiments using subsoil covered by recent volcanic ash (Yamanaka and Okabe 2006) and soil denuded by a landslide (Yamanaka et al. 2022). In the latter study, four ECM morphotypes, including *Alpova*, were confirmed in naturally established seedlings on the denuded soil. These studies indicate that soil spore banks of *Alpova* had developed before the eruption and remained alive underneath the volcanic substrates. Yet we still do not know what ECM fungal species appear at the early primary succession on raw volcanic substrates and how ECM fungal communities change during succession.

In the present study, we investigated ECM fungal communities of *Alnus sieboldiana* at different host growth stages during primary succession at a volcanic site on Izu-Oshima Island, Japan. This pioneer tree species is endemic to Japan and mainly inhabits the Izu Islands in southern Japan. This study aimed to clarify the earliest successional pattern of the *Alnus* ECM fungal community on the volcanic substrate at different host growth stages. We also discuss the similarities and differences in ECM fungal succession between *Alnus* and previously known *Pinus* and *Salix*, and their potential impacts on vegetation succession.

## Materials and methods

### Study sites and field sampling

This study was conducted on Izu-Oshima Island, located about 110 km southwest of Tokyo, Japan. Izu-Oshima Island belongs to a warm temperate zone with an annual mean temperature of 16.4 °C and annual mean precipitation of 2858.9 mm. Mt. Mihara (758 m above sea level), located at the center of the island, is an active volcano that last erupted in 1986. The eastern side of Mt. Mihara has become a volcanic desert covered with scoria and volcanic ash deposited by the last eruption. Three ECM tree species (*A. sieboldiana*, *Castanopsis sieboldii*, and *Pinus thunbergii*) are distributed within Izu-Oshima Island, while only alder (*A. sieboldiana*) was observed near the desert. Since the last eruption, the vegetation has been recovering from the surrounding areas, but a large part remains bare ground. Non-mycorrhizal *Fallopia japonica* var. *hachidyoensis* and *Carex oshimensis* were sporadically distributed at the forefront of invasion toward the bare ground, followed by *A. sieboldiana* and *Weigela coraeensis* var. *fragrans*. Vegetation patches were only observed near the surrounding forests (< 50 m). These patches were composed of multiple plant species, including *A. sieboldiana*, *Angelica keiskei*, *Astilbe hachijoensis*, *Carex doenitzii* var. *okuboi*, *C. oshimensis*, *F. japonica* var. *hachidyoensis*, *Miscanthus condensatus* var. *sieboldii*, and *W. coraeensis* var. *fragrans*. Forests surrounding the volcanic desert were mainly composed of *A*. *sieboldiana*, *Eurya japonica*, *Ilex crenata* var. *hachijoensis*, and *W. coraeensis* var. *fragrans*, where alder was the only ECM host.

Roots of *A. sieboldiana* were sampled in June 2021 over an area of about 8 ha from the forest edge to the bare ground in the southern part of the volcanic desert. To compare the ECM fungal communities among host and vegetation developmental stages, we divided the samples into the following four stages based on host size and accompanying plants: stage 1, seedlings (< 10 cm in height) established solitarily on bare ground; stage 2, saplings (> 10 cm in height, forming the crown) without any accompanying plants; stage 3, saplings in vegetation patches; and stage 4, mature trees at the forest edge. We sampled 30 seedlings and their entire root systems. From stages 2 to 4, we traced the roots and sampled three replicate lateral root systems (~ 15 cm long) from each of the 30 saplings or mature trees per stage. The sampling points were at least 5 m apart, and their geographical positions were recorded by GPS (Garmin 62S; Garmin International, Olathe, KS, USA). We also measured the host’s height and long and short crown diameters to calculate the crown size by an approximate ellipse. The collected samples were placed separately in plastic bags and stored at 4 ℃ until use.

### Root tip sampling and molecular analyses

Each seedling was separated into aboveground and belowground parts. The numbers of ECM root tips and root nodule lobes in the belowground parts were counted before morphotyping and ECM sampling as described below. The dry weights of the remaining belowground part after ECM sampling and the aboveground part were measured after oven-drying (70 °C, 24 h).

Root samples were carefully cleaned of soil and debris with tap water. ECM root tips were classified into morphotypes using a dissecting microscope based on their surface color, shapes, texture, and emanating hyphae (Agerer 1991). For each morphotype per each sample, one to three root tips were collected in separate 2.0-mL tubes and stored at − 30 °C until use. We extracted DNA from each ECM root tip using the cetyltrimethylammonium bromide method (Nara et al. 2003b). The internal transcribed spacer (ITS) regions of rDNA were amplified by polymerase chain reaction (PCR) using the forward and reverse primers ITSOF (5'-acttggtcatttagaggaagt-3') and ITS4B (5'-caggagacttgtacacggtccag-3'), respectively (Gardes and Bruns 1993; Tedersoo et al. 2008). For some samples that could not be amplified, another reverse primer—ITS4 (5'-tcctccgcttattgatatgc-3')—was used (White et al. 1990). PCR amplification was performed using an Emerald Amp PCR Master Mix Kit (Takara Bio, Shiga, Japan) under the following conditions: 30 cycles of 98 °C for 10 s, 56 °C for 30 s, and 72 °C for 60 s. The products were purified using ExoSAP-IT (Applied Biosystems, Foster City, CA, USA) and subjected to direct sequencing on a 3730xl DNA Analyzer (Applied Biosystems). Direct sequencing was performed using a BigDye Terminator v3.1 Cycle Sequencing Kit and the primer ITS1 (5'-tccgtaggtgaacctgcgg-3') or ITS4.

Sequences were trimmed and corrected manually using Sequence Scanner Software 2 (ver. 2.0; Amplified Biosystems) and ATGC ver. 7 (Genetyx, Tokyo, Japan). Subsequently, ≥ 250-bp high-quality sequences were assembled into operational taxonomic units (OTUs) based on ≥ 97% and ≥ 98.5% identity in the VSEARCH program (Rognes et al. 2016) because the most frequently used 97% threshold may not be enough to resolve species-level identity and some researchers recommend 98.5% threshold (Nilsson et al. 2019). The taxonomic identity of assembled consensus and unconnected sequences with ≥ 350 bp was assigned based on the results of BLAST searches against the International Nucleotide Sequence Database Collaboration (INSDC) and UNITE (Kõljalg et al. 2005) databases. The ITS sequences identified were deposited in the DNA Data Bank of Japan under accession numbers LC739392–LC739400.

### Soil analysis

Soil samples were air-dried at room temperature and passed through a 2-mm sieve. The soil pH and electrical conductance (EC) were measured after soil suspension in Milli-Q water (Millipore, Billerica, MA, USA) at a 1:5 ratio using a LAQUAtwin Compact pH Meter and EC Meter (HORIBA, Kyoto, Japan). Total C and total N were measured with a Flash EA 1112 CN Analyzer (AMCO, Tokyo, Japan) after the air-dried soil samples were crushed into fine particles and homogenized with a bead beater using a zirconia ball in a 2.0-mL tube.

### Data analysis

Data analyses were performed using R ver. 4.1.2 (R Core Team 2022) unless otherwise specified. Soil properties and host tree size were compared among host growth stages using a one-way analysis of variance and Tukey’s tests. Before performing those analyses, pH, EC, total C, total N, tree height, and crown size (calculated from an approximate ellipse based on the long and short diameters) were log-transformed to reduce variance heterogeneity. The correlation between seedling dry weight and the number of *Frankia* root nodule lobes, or ECM root tips was analyzed by a generalized linear model (GLM) with gamma and Poisson distributions of errors for the dry weight and the number of nodules, respectively, and log link functions for both. We also analyzed the correlation between the number of nodules and ECM tips on each seedling by the same GLM with a Poisson error structure for both variables and the number of fine roots of each seedling included as an offset in the model. 

The Chao2 richness estimators for ECM fungi were calculated for each host growth stage using Estimate S ver. 9.1 (Colwell et al. 2012) with 1000 randomizations. Shannon’s and Simpson’s diversity indices for ECM fungal communities were calculated for each host growth stage using the iNEXT package (Hsieh et al. 2016). For community analyses, Raup-Crick dissimilarity between the host growth stages was calculated from the presence/absence of data on ECM fungal OTUs for each host individual after removing singletons and doubletons. Raup-click dissimilarity is less susceptible to inappropriately different alpha diversity among samples than other major dissimilarity indices such as Jaccard's and Sørensen's dissimilarity (Vellend et al. 2007). Non-metric multidimensional scaling was used to visualize community dissimilarity with 999 permutations in the vegan package (Oksanen et al. 2022). Statistical differences in community composition among host growth stages soil properties, and host size were tested by a permutational multivariate analysis of variance (PERMANOVA; adonis2 function in the vegan package). Pairwise comparisons among host growth stages with a Bonferroni *p*-value adjustment were conducted using the RVAideMemoire package in R (Maxime 2022).

Community nestedness among host growth stages was tested using nested overlap and decreasing fill (NODF) analysis (Almeida-Neto et al. 2008). The data matrix filled with the occurrence data of OTUs per host growth stage was used for NODF analysis. The significance of NODF was tested against 9999 randomly generated matrices (Monte Carlo procedure) under the setting of both column and row totals fixed with a constrained null model (oecosimu function in the vegan package) (Gotelli 2000).

To assess the global distribution and host specificity of the ECM fungal OTUs detected in this study, we explored previous records of similar sequences worldwide. Sequences with ≥ 98.5% identity to our OTUs were retrieved from the INSDC and UNITE databases. Sampling locations and associated hosts of the retrieved sequences were obtained from the annotation information or original publications. Sequences without sampling locations and isolation sources were excluded from the analysis.

## Results

Soil properties such as pH, EC, total C, and total N were not significantly different from stages 1 to 3; however, the soil properties at stage 4 (mature tree stage) differed significantly from those at the other host growth stages (Table 1). The soil pH at stage 4 was significantly lower than in other stages. The soil total N level was extremely low at stages 1 to 3, ranging from 0.008–0.011%. Host crown size and height were significantly different among the stages, except between stages 2 and 3, both of which were sapling stages but with or without accompanying vegetation.

**Table 1:** Soil and host conditions and diversity of ECM fungi at each host development stage. Different letters (a, b, c) indicated significant differences among stages, according to Tukey’s test. *p* < 0.05

Stage	1	2	3	4
Soil and host condition				
pH	5.6 ± 0.2^a^	5.7 ± 0.2^a^	5.8 ± 0.2^a^	5.4 ± 0.3^b^
EC^d^ (µS/cm)	12.1 ± 1.7^a^	13.2 ± 1.8^a^	15.1 ± 3.6^a^	47.3 ± 39.1^b^
Total C (%)	0.230 ± 0.094^a^	0.374 ± 0.188^a^	0.339 ± 0.200^a^	4.450 ± 5.430^b^
Total N (%)	0.008 ± 0.004^a^	0.014 ± 0.012^a^	0.011 ± 0.007^a^	0.330 ± 0.410^b^
Crown size (m^2^)	0.004 ± 0.004^a^	1.556 ± 1.14^b^	1.699 ± 1.83^b^	36.753 ± 31.785^c^
Height (cm)	3.5 ± 1.6^a^	21.1 ± 6.3^b^	37.1 ± 20.6^b^	237.5 ± 109.1^c^
Distance from forest edge (m)	119 ± 21.5^a^	95.7 ± 21.6^a^	39.9 ± 23.5^b^	-
Diversity of ECM fungi				
No. of ECM root tips sequenced	99	315	213	180
Total ECM fungal OTUs	3	7	7	8
ECM fungal OTUs per individuals ± SD	1.1 ± 0.3^ab^	1.9 ± 0.8^c^	1.4 ± 0.7^ab^	1.6 ± 1.0^abc^
Chao2 ± 95% CI	3.0 ± 0.16	7.48 ± 1.26	7.48 ± 1.26	8.0 ± 0.16
Shannon's index ± 95% CI	2.16 ± 0.30	4.24 ± 0.52	4.57 ± 0.68	5.85 ± 0.96
Simpson's index ± 95% CI	1.76 ± 0.32	3.63 ± 0.43	3.67 ± 0.53	4.76 ± 0.97

Values on soil and host properties are mean  ±  SD

^d^*EC* electrical conductance

**Fig. 1:**
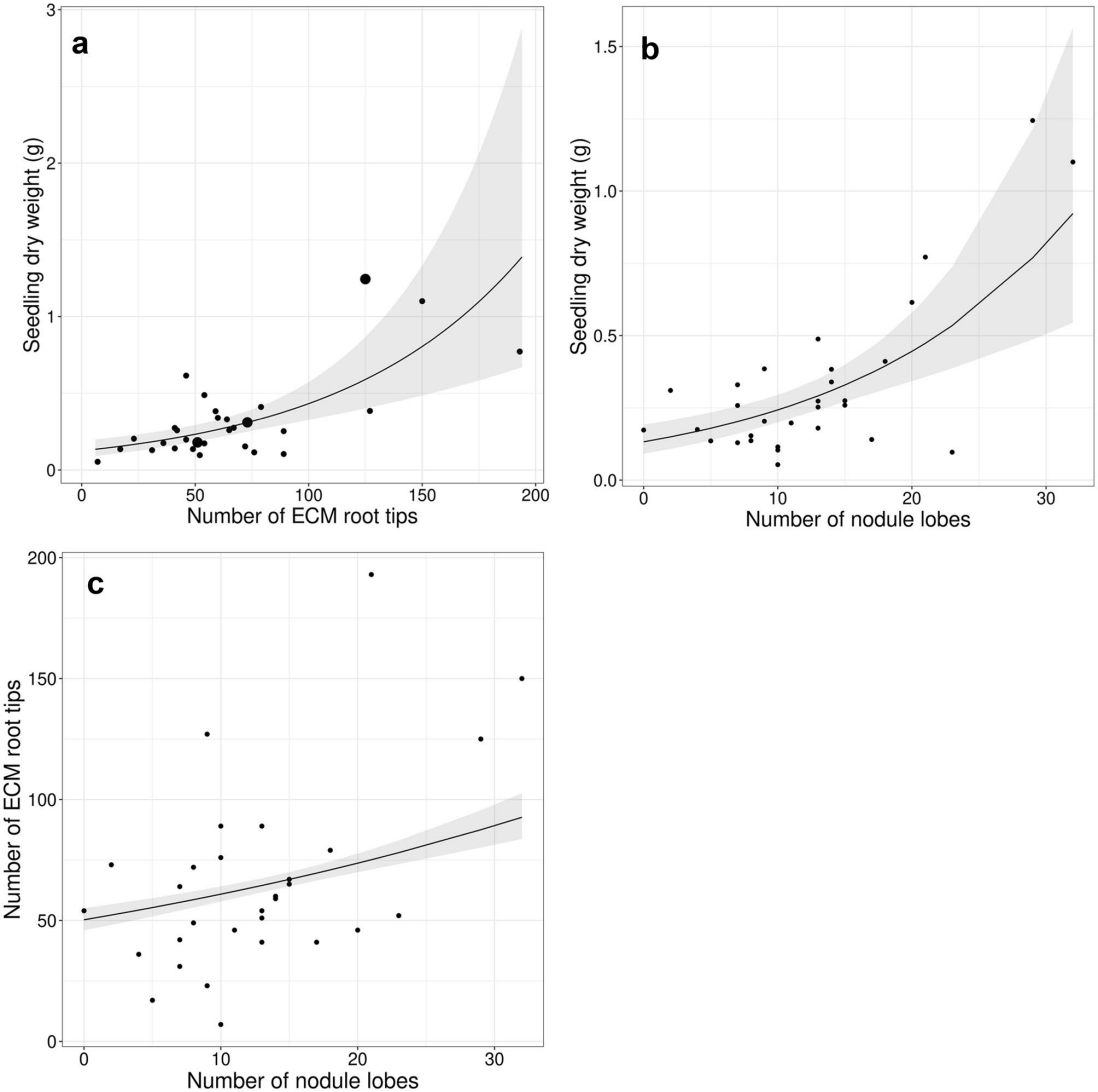
The positive correlation among ECM colonization, *Frankia* nodules, and seedling growth. **a** correlation between the dry weight of *Alnus sieboldiana* seedlings and the number of ECM root tips per seedling (df = 28, t = 4.661, *p* < 0.001). Small dots represent seedlings colonized by *Alpova* sp. alone, while larger ones are colonized by *Alpova* sp. and *Tomentella* spp. simultaneously. **b** correlation between the dry weight of *A. sieboldiana* seedlings and the number of root nodule lobs (df = 28, t = 4.867, *p* < 0.001). **c** correlation between the number of ECM root tips and root nodule lobs per seedling (df = 28, z = 6.983, *p* < 0.001). Shaded areas represent 95% confidence intervals

 ECM root tips were observed in all of the sampled seedlings at stage 1, with an average of 66 ECM tips per seedling. The number of ECM root tips per seedling was significantly correlated with seedling dry weight (Fig. 1a, *p* < 0.001). *Frankia* root nodules were found in 29 of 30 seedlings at stage 1. As in the case of ECM root tips, the number of nodule lobes per seedling showed a significant positive correlation with seedling dry weight (Fig. 1b, *p* < 0.001). We also found a significant positive correlation between the number of ECM root tips and root nodule lobs (Fig. 1c, *p* < 0.001). The root nodules were also confirmed from 28, 27, and 29 host individuals at host growth stages 2, 3, and 4, respectively.

After morphotyping, we collected 99, 315, 213, and 180 ECM root tips at host growth stages 1, 2, 3, and 4, respectively, for DNA extraction (Table 1). We sequenced these 807 ECM root tips, from which 696 high-quality sequences (≥ 250 bp) were obtained. Thirty-three sequences belonged to non-ECM taxa and were thus excluded from the following analyses. Because no high-quality sequences of ECM fungi were obtained from six trees at stage 4, these trees were excluded from the subsequent analyses. Eventually, 663 ECM fungal sequences were clustered into 9 OTUs at both the 97% and 98.5% identity thresholds  (Table 2). Five of the nine OTUs belonged to *Tomentella*, which was the most species-rich genus in this study. Across the host stages, *Tomentella* sp. 1 was the most frequent species with 50% occurrence frequency and was found in 57 of 114 host individuals, followed by *Alpova* sp. with 44% occurrence frequency (50/114 host individuals) (Table 2).

**Table 2:** Species identities, frequency, and global distribution of ECM fungi associated with *A. sieboldiana*

	Acc. No.	Best blast match		Query	UNITE	Stage				Total	Records in other regions
		Acc. No.	Identity	Length	Species hypothesis	1	2	3	4		(With ≥ 98.5% ITS identity)
*Alnicola* sp.	LC739392	HE979487	99.03	515	SH1563786.08FU		2	3		5	Eu, NA, Ja
*Alpova* sp.	LC739393	LC646347	100	664	SH1548909.08FU	24	16	8	2	50	Ja
*Hebeloma submelinoides*	LC739394	LC646348	100	511	SH1563878.08FU		3	2	2	7	Eu, As, Ja
Inocybaceae sp.	LC739395	HE979396	96.79	370	SH1524175.08FU				1	1	
*Tomentella* sp. 1	LC739396	DQ195590	99.13	693	SH1502188.08FU	4	24	18	11	57	Eu, NA, CA, SA, As Ja, NZ
*Tomentella* sp. 2	LC739397	LC646350	100	623	SH1502190.08FU	5	11	10	5	31	Eu, NA, As, Ja
*Tomentella* sp. 3	LC739398	HE979491	100	594	SH1528433.08FU		1	1	12	14	Eu, NA, CA, As, Ja
*Tomentella* sp. 4	LC739399	HE979145	99.83	601	SH1502739.08FU		1	1	4	6	Ja
*Tomentella* sp. 5	LC739400	HE979505	100	505	SH1528404.08FU				2	2	Eu, NA, As, Ja

*Eu* Europ, *NA* North America, *CA* Central America, *SA* South America, *As* Asia, *Ja* Japan, *NZ* New Zealand

**Fig. 2:**
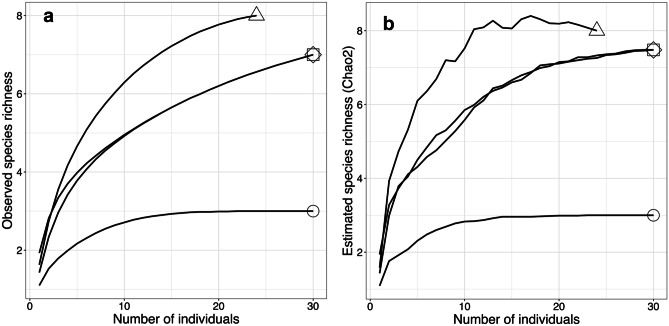
Sample-based rarefaction curves for ECM fungi at each host growth stage. Symbols indicate the host growth stages: stage 1 (circle), stage 2 (square), stage 3 (diamond), and stage 4 (triangle). **a** Observed species richness. **b** Species estimated by the Chao2 estimator

In total, 3, 7, 7, and 8 ECM fungal species were identified from host growth stages 1, 2, 3, and 4, respectively (Table 2). The rarefaction curves for observed ECM fungal species in this study reached a plateau for stage 1 but not for later stages (Fig. 2a). The Chao2 richness estimator was also significantly lower at stage 1 than at later stages (Fig. 2b). Shannon’s and Simpson’s diversity indices also indicated that the alpha diversity of ECM fungi was higher in later host growth stages (Table 2).

**Fig. 3:**
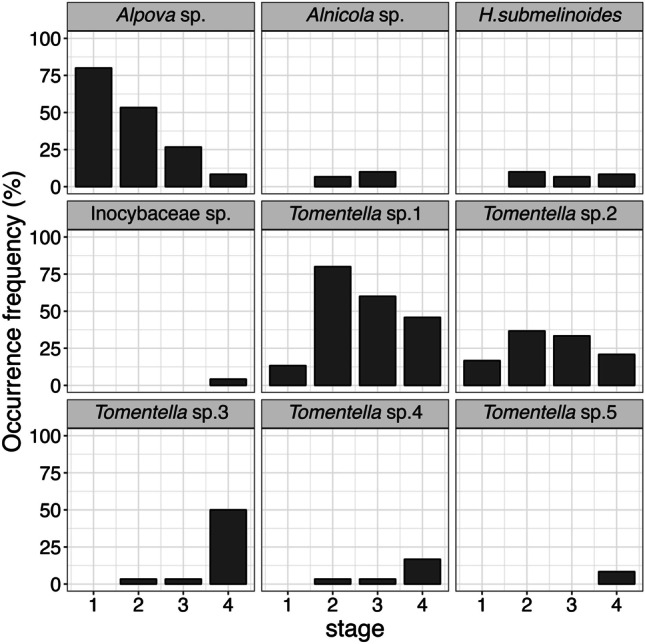
The occurrence frequency of ECM fungi at each host growth stage

Of the three ECM fungal species confirmed on pioneer seedlings at stage 1, an *Alpova* sp. was exclusively dominant with 80% occurrence frequency (24/30 seedlings), followed by *Tomentella* sp. 2 (17%) (Fig. 3). Although the *Alpova* sp. was detected at all stages, the occurrence frequency decreased linearly along the host growth stages. *Tomentella* sp. 1 and *Tomentella* sp. 2 were also detected at all stages, but the maximum frequency of these species was at the sapling stage (stage 2 or 3). In particular, *Tomentella* sp. 1 was the most dominant species in stages 2 and 3, with 80% and 60% occurrence frequency, respectively (Fig. 3).

The most frequent species on mature trees (stage 4) was *Tomentella* sp. 3, with an occurrence frequency of 50%, followed by *Tomentella* sp. 1 (46%) (Fig. 3). Both *Tomentella* sp. 3 and *Tomentella* sp. 4 had the maximum occurrence frequency at the mature tree stage (stage 4), while both species also appeared at the sapling stages (stages 2 and 3) with low frequencies. *Alnicola* sp. and *Hebeloma submelinoides* were found from stage 2 to later stages as minor components. Inocybaceae sp. and *Tomentella* sp. 5 were only detected from stage 4 as a singleton and doubleton, respectively (Table 2).

**Fig. 4:**
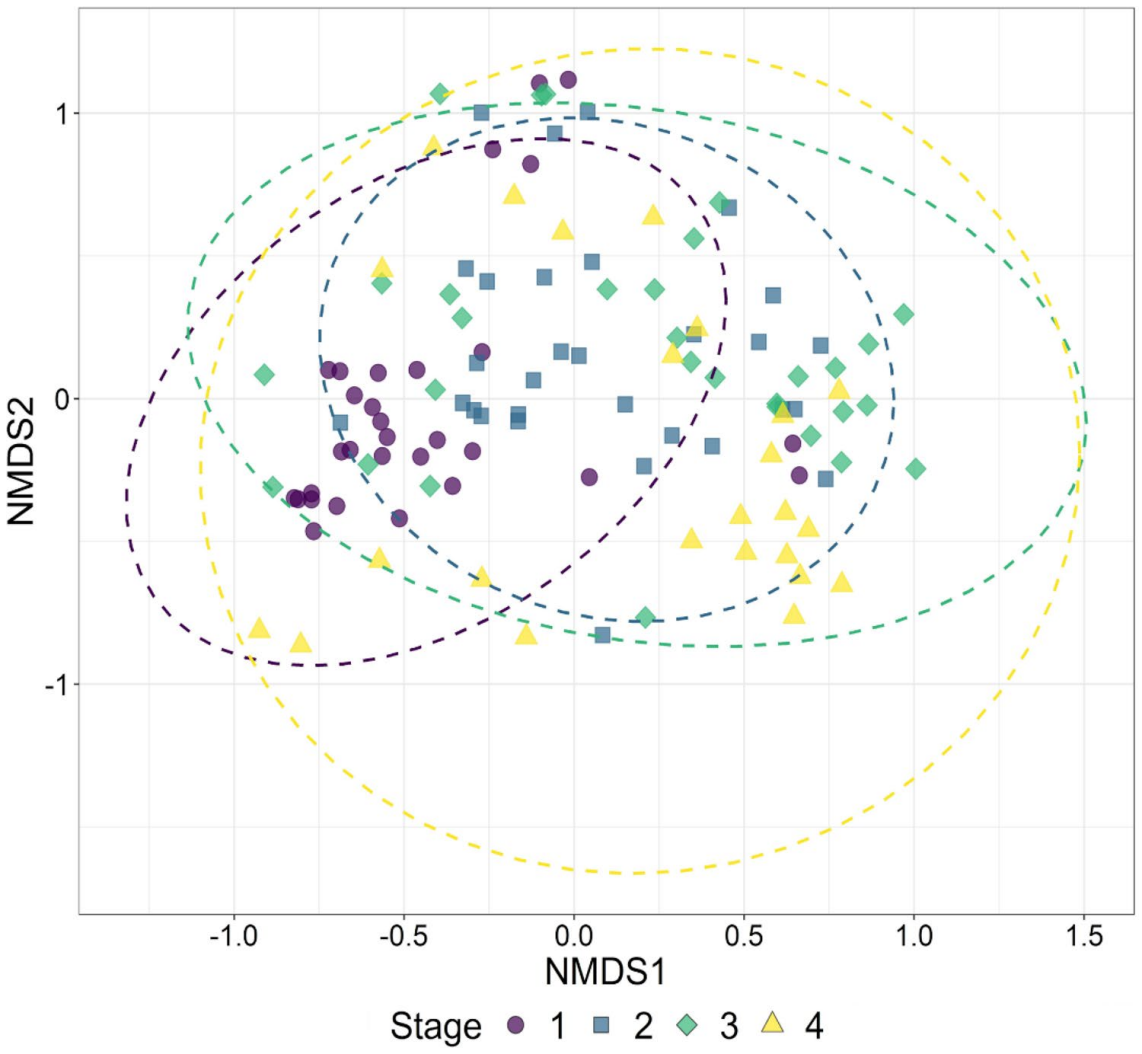
Non-metric multidimensional scaling ordination of the ECM fungal communities at each host growth stage, with respective 95% confidence ellipses. Stress = 0.0648. A small amount of jitter was introduced to the plot to separate overlapping points

The compositions of the ECM fungal communities differed significantly among host growth stages (PERMANOVA: *p* < 0.05), while no significant effect was found in any soil properties examined. Pairwise comparisons of the compositions also revealed significant differences in all pairs of growth stages except between stages 2 and 3 (Table 3). The ECM fungal communities of earlier host stages were clearly nested within those of later host stages (Fig. 4), as confirmed by the significant nestedness index (NODF = 75.5, *p* < 0.05).

**Table 3:** Bonferroni corrected *p* values for all pairwise comparisons of ECM fungal communities among host development stages using a PERMANOVA on a distance matrix

	Stage 1	Stage 2	Stage 3
Stage 2	0.006	-	-
Stage 3	0.006	1	-
Stage 4	0.006	0.006	0.042

We found 321 previous sequence records for 8 of the 9 ECM fungal species identified in this study at the 98.5% ITS identity threshold (Table S1). Almost all of them were obtained from ECM root tips with *Alnus* spp. or sporocarps in *Alnus* forests across Europe, East Asia, and North America. *Tomentella* sp. 1 had the broadest distribution range, including Europe, East Asia, North America, Middle America, South America, and New Zealand. By contrast, previous sequence records for the *Alpova* sp. and *Tomentella* sp. 4 were restricted to Japan. In particular, the previous sequence record for the *Alpova* sp. came from ECM root tips of *A. sieboldiana* on Izu-Oshima Island.

## Discussion

This study provides a new example of primary succession of ECM fungi using pioneer *Alnus* at a volcanic site, broadening our knowledge of ECM fungal succession obtained from other pioneer host genera (*Salix* and *Pinus*) (Jumpponen et al. 2002; Nara et al. 2003b; Ashkannejhad and Horton 2006). The initial ECM fungal community on *Alnus* seedlings was exclusively dominated by an *Alpova* sp. (Fig. 3), indicating its important role at the seedling establishment stage. *Alpova* is specific to alder trees (Molina 1981) and produces hypogeous sporocarps (Molina 1979). Because *Alpova* is known to develop soil spore banks (Miller et al. 1992), ECM colonization of pioneer alder seedlings may largely depend on the spore banks developed after the last eruption. The frequency of the *Alpova* sp. decreased as the host growth stage advanced, specifically to ~ 5% at the mature tree stage, comparable to mature alder stands (Tedersoo et al. 2009; Põlme et al. 2013; Pozzi et al. 2018). Interestingly, these traits of *Alpova* are similar to those of pine-specific *Rhizopogon*, which produces hypogeous sporocarps, develops soil spore banks, appears as a primary colonizer on pioneer pine seedlings (Peay et al. 2007), and decreases with host age (Bruns et al. 2002).

As in most hypogeous fungal species, the *Alpova* sp. likely depends on land animals for spore dispersal (Johnson 1994; Nouhra et al. 2005). However, our research site was located on an oceanic island that has never been connected to any continent or mainland Japan, indicating that dispersal by land animals was impossible. Although we do not know how the *Alpova* sp. or its ancestor initially arrived at Izu-Oshima Island, migrating birds may have dispersed the spores as deposits in their feces, as demonstrated for other fungal species (Caiafa et al. 2021). In addition, because previous sequence records of *Alpova* sp. were only found from Izu-Ohshima Island (Yamanaka et al. 2022) and not in any other geographical regions, this species may have evolved locally on this island, or in close neighboring areas of Japan, over a long period.

*Alpova* forms extensive rhizomorphs (Agerer 2006) and has been shown to improve the phosphorous absorption and growth of seedlings when combined with another symbiotic partner (*Frankia*) in an in vitro experiment (Yamanaka et al. 2003). In the present study, both ECM colonization (mostly *Alpova* sp.) and Frankia nodule formation were significantly correlated with seedling dry weight, indicating that these microbes could improve host growth in the field. Therefore, a tripartite symbiosis—namely, *Alnus*, *Alpova*, and *Frankia*—may be the key to successful alder establishment on the extremely nutrient-poor volcanic substrate at this primary successional site.

In contrast to the *Alpova* sp., most of the ECM fungi detected in this study had a Holarctic or global distribution confirmed by previous sequence records in international databases. Such wide distribution ranges indicate efficient spore dispersal by wind (Peay et al. 2011; Nouhra et al. 2015). Moreover, all of these previous sequence records were obtained exclusively from alder stands (Pritsch et al. 2010; Põlme et al. 2013; Yamanaka et al. 2022). The strong preference of alder for specialist ECM fungi may reduce competition with other local ECM fungi and facilitate comigration when exploring new habitats.

As in primary successions of ECM fungi on other host species, the diversity of ECM fungi increased with the host developmental stage by recruiting new species without replacing pre-existing species (Nara et al. 2003b; Ashkannejhad and Horton 2006). The nested structure of ECM fungal communities documented in other hosts (Peay et al. 2007) was confirmed in this study. The apparent difference from other pioneer hosts is that the total richness of ECM fungal diversity at the mature tree stage was far lower in alder than in other host species. The lower ECM fungal diversity on alder has been previously reported from mature stands (Tedersoo et al. 2009; Kennedy and Hill 2010; Bent et al. 2011) and is probably related to the nitrogen-fixing ability of the alder, as nitrogen availability has a significant impact on ECM fungi (Kennedy et al. 2015). Another interesting finding is that the richness of the ECM fungi at the sapling stages (seven species observed) was nearly the same as that at the mature tree stage (eight species). Such saturation of ECM fungal diversity at the sapling stages may be unique to alder hosts and related to the small ECM fungal pool within the oceanic island. 

The observed dominance of specialist ECM fungi on alder throughout host development under primary settings is quite different from pioneer willow and pine hosts, both of which are mainly associated with generalist ECM fungi at later host developmental stages (Nara et al. 2003b; Ashkannejhad and Horton 2006). The existence of generalist ECM fungi on pioneer hosts directly facilitates ECM colonization and the establishment of other subsequent host species by providing compatible ECM fungal inocula or forming mycelial networks (Nara and Hogetsu 2004). Thus, the absence of generalist ECM fungi in this desert would not directly facilitate the establishment of other ECM hosts and could even inhibit their establishment due to the pre-existing incompatible mycelial network (Kropp and Trappe 1982; Kennedy et al. 2015). In fact, we did not observe any other ECM trees established near alder trees in this volcanic desert. Furthermore, ECM alder forests are replaced by arbuscular mycorrhizal forests composed mainly of *Prunus speciosa* and *Machilus thunbergii*, and then converge into ECM climax forests dominated by *C. sieboldii* during typical vegetation succession on the Izu Islands (Kamijo et al. 2002). Although diverse ECM fungi have been recorded from *C. sieboldii* forests even on the neighboring Hachijo Island (Miyamoto et al. 2018), none of them were shared with alder trees in this study. Therefore, a direct effect of pioneer alder on vegetation succession through ECM fungi may be unlikely, although the nitrogen input by alder trees would affect vegetation succession (Hobbie et al. 1998; Titus 2009).

## Supplementary Information

Below is the link to the electronic supplementary material.

Supplementary file1 (XLSX 35 KB)
