# Correction to: Primary succession of ectomycorrhizal fungi associated with Alnus sieboldiana on Izu-Oshima Island, Japan

**DOI:** 10.1007/s00572-023-01116-6

**Published:** 2023-06-22

**Authors:** Akira Ishikawa, Kazuhide Nara

**Affiliations:** Department of Natural Environmental Studies, Graduate School of Frontier Sciences, The University of Tokyo, 5-1-5 Kashiwanoha, Kashiwa, Chiba 277-8563 Japan

**Correction to: Mycorrhiza (2023) 33:187–197** **https://doi.org/10.1007/s00572-023-01112-w**

The original published version of the above article contained errors. The following data should have been corrected as follows:

1. The first Fig. 1 citation should have been Fig. 1a
2. The text below under Result section should have been corrected as follows:
  The text should be "occurrence" instead of "relative"
  The text should be "subsequent" instead of "community"
  The text should be "alpha" instead of "beta"
  The text should be "80% and 60% occurrence" instead of "41% and 42% relative"
  The text should be "frequent" instead of "dominant"
  The text should be "an occurrence frequency of 50%, followed by Tomentella sp. 1 (46%)" instead of "a relative frequency of 31%, followed by Tomentella sp. 1 (28%)"
3. The text below under Discussion section should have been corrected as follows
  The text should be "age" instead of "growth"
  The text should be "dispersal" instead of "migration"
  The text "indirectly" should be deleted

The original article has been corrected.

